# Interpreting the results of noninferiority trials—a review

**DOI:** 10.1038/s41416-022-01937-w

**Published:** 2022-09-15

**Authors:** Jack Cuzick, Peter Sasieni

**Affiliations:** 1 Centre for Cancer Prevention, Wolfson Institute of Population Health, Queen Mary University of London, Charterhouse Square, London, EC1M 6BQ UK; 2 School of Cancer & Pharmaceutical Sciences, Faculty of Life Sciences & Medicine, King’s College London, London, UK

**Keywords:** Risk factors, Medical research

## Abstract

Noninferiority trials are becoming increasing common, but are often poorly reported and misunderstood. A better understanding of the new components of a noninferiority trial and their interpretation is needed. Noninferiority trials are an extension of conventional superiority trials, which provide a basis for determining if a new treatment, which may have advantages other than efficacy, has sufficient efficacy to be useful in certain situations. A key feature is the need to specify a clinical noninferiority margin above which the lower boundary of the confidence interval for the difference between the new treatment and the conventional treatment must lie. In most cases a nontreated control arm is not included, and when the efficacy of the new treatment is less than that of the standard treatment, determining its efficacy versus no treatment can be a major challenge. Treatments meeting a clinical noninferiority requirement can be statistically significantly superior to standard treatment, of similar efficacy (i.e., no significant difference), or even significantly inferior in a conventional analysis. Noninferiority comparisons are an important addition to the reporting of clinical trials, but require prior consideration of several factors that conventional superiority analyses do not address.

## Introduction

Noninferiority outcomes were developed to determine if a new treatment might be at most marginally less efficacious than the standard treatment. This is particularly relevant in assessing bioequivalence of a new formulation to an established drug. When other aspects of the treatment are also important in determining its use, a treatment that is, say, 90% (or even 50%) as effective as the standard treatment might be considered of value in certain circumstances. An example is when the new treatment may provide safety advantages compared with the standard treatment, so that slightly lower efficacy may be acceptable especially so if the disease is rarely fatal, and the new treatment is less toxic or cheaper. In these cases, a noninferiority analysis can complement the traditional approach—where a new treatment is considered superior to the conventional treatment only if the 95% confidence interval for the difference is completely in the positive effect size region, inferior if it is completely in the negative region, and inconclusive if it straddles the no effect point.

Noninferiority extends these possibilities by determining if the new treatment is “not substantially worse” than the standard treatment based on the confidence interval of this difference not including a predefined unfavourable difference in outcomes—the noninferiority margin. In the literature, noninferiority has sometimes been used when the difference between two treatments is inconclusive without reference to a noninferiority margin. Such use is inappropriate. An inconclusive outcome simply means that no statistically significant difference was seen between the two treatments. If the trial was underpowered the actual difference might still be substantial. It is important to recognise that a further requirement for inferiority is that the confidence interval excludes the predefined noninferiority margin. This guarantees that with high confidence (usually 95%) the new treatment is not substantially worse than the standard treatment (i.e., not worse by as much as the predefined amount). Thus for a noninferiority margin of 10%, a treatment that was 3% worse with a 95% confidence interval of 15% worse to 9% better cannot be said to be noninferior (because it could be as much as 15% worse). However, if the confidence interval was from 7% worse to 1% better, it would be considered noninferior for this noninferiority margin, even though it is statistically significantly worse (the 95% confidence interval excludes no difference). Thus, while an inconclusive finding does not directly provide any information on the performance of the two treatments, a noninferior finding implies that compared with the standard treatment, the new treatment is at most worse by no more than a predefined amount. If the new treatment has other benefits, such as fewer side effects, lower cost, or ease in delivery, this may be acceptable.

**Fig. 1:**
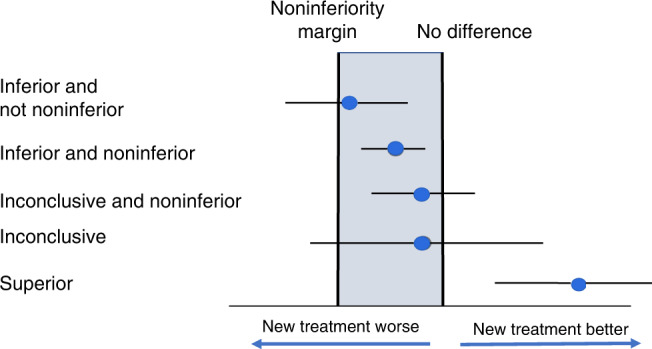
Treatment effect and 95% confidence interval. Point estimate and 95% confidence interval for different outcomes using conventional and noninferiority methods.

Here, we focus on the specific issue of understanding the limitations and meaning of noninferiority results. In particular, we explore situations where a ‘noninferior treatment’ based on a specific choice of a noninferiority boundary can be traditionally inconclusive, traditionally superior, or, indeed, traditionally inferior to the conventional treatment (Fig. 1). We also discuss the need for additional complementary analyses, based on assessing the efficacy of the new treatment compared with no treatment. We recommend that two margins be set—one for clinical noninferiority against the standard treatment and another for efficacy relative to no treatment and that both must be satisfied before accepting a new treatment.

## Observations

Several new treatments have been appropriately accepted as useful based on noninferiority trials [1, 2].

**Fig. 2:**
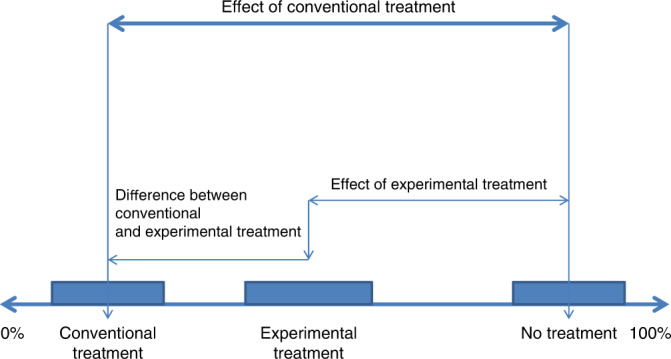
Percentages of patients with a recurrence (with confidence intervals) according to a treatment option. Efficacy endpoints in noninferiority studies. Results for three groups need to be considered—a group receiving the conventional treatment, one receiving the experimental treatment and an untreated group. The experimental treatment needs to be evaluated against the conventional treatment, and if not better, also a no-treatment group. Within a standard two-arm noninferiority trial, only the difference between the conventional and experimental arms can be directly measured. The difference between the conventional treatment and no treatment needs to be inferred from historical data, and these two are then used to estimate the difference between the experimental treatment and no treatment.

A full analysis of a noninferiority trial requires consideration of three outcomes – one each for an untreated population, the population receiving the standard treatment, and the population receiving the experimental treatment (Fig. 2). In contrast, a conventional analysis only requires a comparison of two event rates—the standard established treatment (or no treatment when no established standard treatment exists) against the new treatment. Noninferiority analyses focus on the difference between the conventional treatment and new treatment, but some (typically historical) evidence is needed to estimate the benefits of the new treatment versus no treatment in the population under study. Superiority to no treatment is not addressed directly by the noninferiority comparison, and this is especially relevant when the point estimate of efficacy for the new treatment is below that of the standard treatment.

The European Medicines Agency (EMA) and the U.S. Food and Drug Administration (FDA) guidelines both recommend that, generally, a 95% two-sided confidence interval be used for assessing noninferiority [3, 4]. Thus, not only must the point estimate of progression rate be above the noninferiority cut-off, but the entire confidence interval must lie within the noninferiority region. These documents and more recent papers [5–8] provide a general discussion of issues related to noninferiority designs, including a review of major papers published in this area [1].

As with conventional superiority trials, one must specify the population being evaluated —typically intention-to-treat (ITT), per protocol or as treated are used. Whereas using ITT may be thought of as conservative in a superiority trial and is generally recommended, it is anti-conservative in a noninferiority trial because protocol violations will make the two arms more similar and hence reduce the chance of finding a difference between the two treatments if one exists. Ideally, a noninferiority trial should be analysed in a way that estimates the causal impact of the treatment, but if that is too complicated, one should consider both the ITT and the per-protocol analyses and require noninferiority to be met for both. Another point to considered with a noninferiority analysis is that the new treatment may be more acceptable to patients than conventional treatment, potentially due to greater ease of use or fewer side effects, leading to a higher compliance rate. Hence it is important to report the compliance with treatment in both arms, and discuss as necessary.

## Noninferiority margin

Determination of the noninferiority margin is the main challenge in designing noninferiority analyses, and its choice is central for drawing meaningful conclusions. The margin should depend on the severity of the endpoint. For example, a smaller margin would be appropriate when mortality is the endpoint, as compared with, say, the need for additional salvage treatment. The margin should also take into account the magnitude of any other benefits of the new treatment. Often this margin is referred to as 'clinically noninferior'. For these reasons, the noninferiority margin should be determined in advance, ideally by a panel of patients, clinicians and researchers and specified in the protocol.

Crucial to this decision is reliable knowledge of 'disease progression' in the absence of treatment, and the benefit achieved with the conventional treatment. Progression can be measured in several ways, including mortality, recurrence, an increase in extent of disease (stage) or severity of symptoms. Consideration should be given as to how many additional cases of 'progression' one would accept for every 100 patients treated with the new versus the standard treatment. Whatever measure is used, it must be predefined and based on prior data, in which the ‘other’ benefits of the new treatment are quantified and used to determine how great any inferiority of the new treatment compared with the standard can be considered acceptable.

Noninferiority can be assessed either as a relative or absolute difference in efficacy between the standard treatment and the experimental treatment, and both have their advantages and disadvantages. The absolute difference is often most relevant in discussions with patients, as it reflects a change in the risk of progression without reference to the overall risk of progression or the reduction associated with standard treatment. The relative difference can be particularly helpful in understanding how the new treatment compares with the conventional treatment in terms of how much of the overall treatment effect compared with no treatment is retained or extended. This is most relevant when the new treatment is less efficacious than the standard, when its efficacy compared with no treatment becomes questionable.

## Comparisons with a no-treatment arm

For a new treatment to be considered acceptable it is essential that it is shown to be superior to no treatment. In most noninferiority trials, this is a problem because there is no untreated (or placebo only) arm; the new treatment is only compared with the accepted conventional treatment. A key challenge is to estimate the counterfactual: what would have happened had the patients not been treated, and consider the potential benefits of the new treatment compared to no treatment. Even if not as efficacious as the standard treatment, it is essential that the new treatment provides some benefit—i.e., it should be superior to a placebo. Assessing this is necessarily based on comparisons with a historical series, and to do this a second ‘efficacy’ margin needs to be specified to indirectly infer superiority to no treatment. Even if guided by a meta-analysis of trials of standard treatment compared with no treatment, the magnitude of this effect will be less reliably determined, as it is necessarily based on an indirect comparison with a historical reference. Using the observed progression rate in an untreated population may provide a poor counterfactual for the population under study if improvements in supportive treatment have led to a better prognosis, or if simply the inclusion criteria of historical trials were quite different from those in the current trial.

To illustrate this point, consider a conventional treatment that historically reduced 5-year recurrence from 30 to 20%, and suppose in the noninferiority trial, that the new treatment leads to 4% more recurrence than the conventional treatment. If, as before, the conventional treatment results in 20% recurrence, this 4% increase translates to a 20% relative (6% absolute) decrease in 5-year recurrence compared with no treatment (assuming recurrence with no treatment would still be 30%). However, if due to changes in other aspects of usual care or the study population, the conventional treatment leads to a 5-year recurrence of just 8% in the new trial and 12% on the new treatment, the question of what the recurrence rate would have been with no treatment becomes critical. If one assumes the benefit of conventional treatment is proportional to the risk of recurrence, then recurrence with no treatment would again be 50% greater, corresponding to 12% and indicating no effect of the new treatment. If, instead, one assumes the absolute benefit of conventional treatment is preserved, recurrence with no treatment would have been 18% (8% + (30–20%)), considerably more than 12% on the new treatment. For simplicity we have illustrated these issues in terms of the point estimates, but in practice it is essential to use the 95% confidence intervals for the treatment effects.

To infer some efficacy of the new treatment versus no treatment, its minimal effect (i.e., the upper limit of the 95% confidence interval for recurrence) versus conventional treatment should be less than the minimal effect of conventional treatment versus no treatment (termed M_1_ by the FDA). As M_1_ is based on historical comparisons, and only supports some efficacy of the new treatment vs no treatment, the FDA recommends taking some fraction of M_1_, e.g. M_1_/2, for the ‘efficacy’ margin so that at least half the efficacy of the conventional treatment is retained [3, 4]. Note that specifying a smaller fraction is a more demanding requirement in that a greater proportion of the benefit of the conventional treatment must be retained, e.g. using M_1_/5 would require that at least 80% of the conventional treatment vs no treatment effect is retained. In many cases, the stipulation based on the noninferiority margin will be more demanding, especially when there is a large and well-established effect of the conventional treatment. When the population being studied has changed in an important way from the historical standard, the estimate of M_1_ can be unreliable. In this situation, there may be a case for a three-arm trial, in which a no-treatment arm is added alongside the conventional treatment and the new treatment arms. This is only justified if there is uncertainty (equipoise) as to whether the conventional treatment is superior to no treatment in this new setting.

## Subgroup analysis

In a superiority trial that includes both good and poor prognosis patients, it is often assumed that a treatment shown to be superior overall will be superior for both good prognosis and poor prognosis patients (provided there is no evidence of an interaction). The same is less likely to be true for a noninferiority comparison. A treatment could be clinically noninferior for good prognosis patients but not for poor prognosis patients for whom the absolute benefit of a more effective treatment is likely to be larger and more easily established. For most treatments, the relative efficacy tends to be more homogenous across different prognostic groups than the absolute efficacy, and thus has the advantage that it is more often applicable to patients with different underlying risks of progression. However, to aid patients in weighing up the pros and cons of two different treatments, it is generally the absolute difference in risk that is most relevant. A comparison of treatments by relative risk can mask important differences in absolute risk for patients with different prognoses. Consider a new treatment that is overall less effective with relative risk of 1.25. For poor prognosis patients with a 20% chance of recurrence on conventional treatment that would translate into an additional 5% chance of recurrence. Whereas, for good prognosis patients with a 6% chance of recurrence on conventional treatment, this implies a 1.5% additional chance of recurrence. On this basis, the new treatment might be considered noninferior for good prognosis patients, but not for poor prognosis patients, in keeping with treating poor prognosis disease more aggressively. Thus, secondary comparisons of the new and conventional treatments in predefined good and poor prognosis patients (on both a relative and absolute scale) is highly desirable. It may be too much to ask to demonstrate noninferiority separately in each subgroup, but it may be appropriate to consider the overall treatment effect (and its confidence interval) applied to the outcome in controls in different subgroups.

Specifying the noninferiority margin for the absolute difference has the additional advantage that it can be more easily compared with other treatment effects. For determining how much loss of efficacy is acceptable when balanced against advantages such as reduced toxicity, convenience, or reduced costs, it is the absolute effect that will enter an overall health economic analysis.

## Inconclusive noninferior treatments

As illustrated in Fig. 1, treatments found to be ‘noninferior’ can be conventionally classified as inconclusive, superior or inferior. This is somewhat confusing language, but many trials have established and reported that a new treatment is noninferior to the conventional treatment, but evidence for superiority or inferiority is inconclusive [1].

A recent example of this is in the treatment of metastatic renal cell carcinoma, where nephrectomy was traditionally used. More recent evidence has supported the addition of a targeted therapy. The CARMENA trial [9] compared nephrectomy in conjunction with the targeted therapy sunitinib versus sunitinib alone and asked if the latter was noninferior to the joint treatment. The noninferiority boundary was that the upper limit of the two-sided 95% confidence interval for the hazard ratio for overall survival should be below 1.20. After a median follow-up of 50.9 months, the hazard ratio for sunitinib treatment alone was 0.89, 95% confidence interval (0.71–1.10). Thus, while the small reduction in deaths with sunitinib alone was statistically inconclusive, the noninferiority margin was met.

Another example concerns hypofractionation of radiotherapy for breast cancer [10]. In the FAST-Forward trial the standard 40 Gy in 15 fractions over 3 weeks was compared with two regimens of five fractions in one week: using either 26 Gy or 27 Gy. Based on historical comparisons, the trialists assumed 2% 5-year incidence of ipsilateral recurrence on the standard regimen and specified a 1.6% noninferiority margin for the differences. After a median of 71.5 months, the 5-year incidence of ipsilateral recurrence was 2.1%, 95% CI (1.4–3.1%) on the 15-fraction 40 Gy schedule. It was 0.7% lower 95% CI (−1.3 to 0.3%) on the 5-fraction 26 Gy schedule; and 0.3% lower 95% CI (−1.0 to 0.9%) on the 27 Gy schedule. Neither of these differences was statistically significant, but for both the 95% confidence interval was within the noninferiority margin (of 1.6%). Thus, each was noninferior, but inconclusive regarding superiority.

## Superior noninferior treatments

By definition, conventionally superior treatments are also noninferior. However, conventional trials typically do not specify a noninferiority boundary, and the issue of wanting to declare superiority from a noninferiority trial is rarely encountered. One nice example of this occurrence is the ATAC trial (Anastrozole, Tamoxifen Alone or in Combination) [11]. This was a three-arm trial in which tamoxifen alone (the standard treatment) was compared with anastrozole alone or tamoxifen and anastrozole in combination in 9366 women with early breast cancer. Two co-primary comparisons were specified, namely: is the combination of tamoxifen and anastrozole better than tamoxifen alone; and is anastrozole alone noninferior to tamoxifen alone. Noninferiority was based on the requirement that the 90% two-sided upper confidence limit of the hazard ratio for disease-free survival of anastrozole vs tamoxifen was less than 1.25. In fact anastrozole alone was significantly better than tamoxifen (*P* = 0.013), so that the noninferiority question was overtaken by the finding of a conventionally superior outcome. In fact, the combination treatment turned out to be slightly worse than tamoxifen, and significantly worse than anastrozole alone (*P* = 0.0006). Superiority of anastrozole to tamoxifen in terms of efficacy has been confirmed in additional long-term follow-up studies extending up to 10 years [12, 13].

## Inferior ‘noninferior’ treatments

A treatment is either noninferior or ‘not noninferior’. Curiously, when clinical noninferiority is established, the new ‘noninferior’ treatment can be inferior to the comparator treatment in the conventional sense of being statistically significantly worse (Fig. 1). This confusing language occurs because a noninferiority comparison allows a noninferiority margin, and if this is made large enough, a treatment which is significantly less efficacious than the standard treatment can still meet the noninferiority criterion. Thus, a full evaluation should also consider if the new treatment is statistically inferior to the conventional treatment. When this is the case, it is particularly important to ask, ‘What is the evidence that the new treatment is more efficacious than no treatment?’ Failure to address these points is a serious omission and can lead to an unbalanced evaluation of the value of the new treatment [2].

Such an outcome arose in the TARGIT-A trial of intraoperative radiotherapy (IORT) for localised breast cancer [14–16]. Here, a 2.5% absolute difference in 5-year local recurrence was the pre-specified noninferiority margin. This noninferiority margin was met [13], but IORT led to more than twice the number of local recurrences when compared with conventional external beam radiotherapy (EBRT), and this difference is conventionally statistically significant (i.e., the null hypothesis of no difference in local recurrence rate would be rejected) for the predefined 5-year follow-up (24 vs 11 cases; 5 y Kaplan–Meier 2.33% vs 1.02%, difference 1.21% (95% CI (0.33–2.09%); binomial RR = 2.22, *P* = 0.024). Further, using all available follow-up (median 8.6 years), the absolute difference between treatments was substantially larger (60 vs 24 cases, 5.26% vs 2.07%, difference 3.19%) and this difference in proportions was highly significant (*P* = 0.00004) [[13], Table 3]. Thus, IORT was noninferior but conventionally inferior to EBRT in terms of 5-year local recurrence. However, in a secondary analysis, the authors report that when all-cause mortality is included for a composite endpoint of local recurrence-free survival, no significant differences were seen between treatment arms ([15], Fig. 3).

IORT could either be superior or non-superior to no treatment. Historically, local recurrence rates were high, and the addition of EBRT reduced 10-year local recurrence by almost two-thirds, from roughly 30–10%, in node-negative disease treated by breast-conserving surgery [17]. In their 2014 overview, Houssami et al. [18] report median local recurrence of 5.3% with a median of 6.6 years follow-up with whole breast radiotherapy. In more recent times, even lower rates have been achieved: 5-year local recurrence in TARGIT-A was just 0.95% in the conventional treatment arm.

This example makes clear that to focus only on absolute differences without a discussion of relative differences can be misleading. Thus, while a 2.5% absolute inferiority margin might have been reasonable when local recurrence was around 6% in patients receiving radiotherapy (i.e., 42% worse), as was the case when the trial was planned, it may not be reasonable when only 1% of conventionally treated patients had local recurrence at 5 years. Here, a rescaling to preserve the relative difference would roughly correspond to a 0.5% absolute noninferiority margin.

## Conclusions and relevance

In summary, care needs to be taken in the use and interpretation of noninferiority trials. Ideally, two margins should be specified: one based on an absolute difference for determining clinical noninferiority compared with standard treatment; and a second based on a relative difference for inferring efficacy relative to no treatment. A comparison with no treatment is essential when the new treatment is noninferior but numerically less efficacious than the standard treatment. This is usually based on meta-analysis of historical comparisons of the conventional treatment with no treatment, focusing on patients with similar prognosis to the population in the current trial.

As with conventional superiority trials, one must specify the population being evaluated. Typically, intention-to-treat (ITT), per protocol or as treated are used. One point that needs to be considered with a noninferiority analysis is that the new treatment may be more acceptable to patients than conventional treatment, potentially due to greater ease of use or fewer side effects, leading to higher compliance. Usually, the ITT population is most appropriate, but as noted above, this can underestimate the actual difference between treatments, and it is important to report the compliance with treatment in both arms and discuss as necessary.

Noninferior treatments can be conventionally inferior, non-significantly different, or superior to the standard treatment, and a conventional analysis also needs to be provided to report this. When the new treatment is inferior, further analysis of the extent of the inferiority, and clear evidence of some other benefit is essential.
